# Socioeconomic inequalities in the utilisation of hypertension and type 2 diabetes management services in Indonesia

**DOI:** 10.1111/tmi.13303

**Published:** 2019-09-15

**Authors:** Joko Mulyanto, Dionne S. Kringos, Anton E. Kunst

**Affiliations:** ^1^ Department of Public Health and Community Medicine Faculty of Medicine Universitas Jenderal Soedirman Purwokerto Indonesia; ^2^ Department of Public Health Amsterdam UMC University of Amsterdam Amsterdam Public Health Research Institute Amsterdam The Netherlands

**Keywords:** inequalities, socioeconomic status, hypertension, type 2 diabetes, healthcare, Indonesia, Inégalités, statut socioéconomique, hypertension, diabète de type 2, soins de santé, Indonésie

## Abstract

**Objectives:**

To describe socioeconomic inequalities in the utilisation of hypertension and type 2 diabetes (T2D) management services in the Indonesian population and to determine whether education level and geographical location contribute to inequalities.

**Methods:**

Cross‐sectional study using data from the 2014 Indonesia Family Life Survey (*N *=* *30 762 for hypertension; *N *=* *6758 for T2D). Socioeconomic status was measured by household consumption. The prevalence of hypertension and T2D was determined using internationally standardised clinical measurement, while disease management was defined by participation in screening and current use of medication. The relative index of inequality (RII) was used to estimate inequalities, adjusted to education level and geographical location.

**Results:**

For all household consumption quintiles, we observed low rates of screening participation for T2D and low medication use in both hypertension and T2D. We found socioeconomic inequalities in screening participation for hypertension (RII 2.68, 95% CI 2.42–2.96) and T2D (RII 7.30, 95% CI 5.48–9.72) and also for medication use in hypertension (RII 3.09, 95% CI 2.28–4.18) and T2D (RII 2.81, 95% CI 1.09–7.27). Education level contributed to socioeconomic inequalities in screening utilisation for both hypertension and T2D. Geographical location contributed to inequalities in screening utilisation and medication use for T2D. Socioeconomic inequalities in medication use for hypertension and T2D were larger among men than women.

**Conclusions:**

Large socioeconomic inequalities were found in the utilisation of hypertension and T2D management services in Indonesia. Improving affordability, availability and approachability of services is crucial to reduce such inequalities.

## Abstract (fr)

**Objectifs:**

Décrire les inégalités socioéconomiques dans l'utilisation des services de prise en charge de l'hypertension et du diabète de type 2 (DT2) au sein de la population indonésienne et déterminer si le niveau d’éducation et la localisation géographique contribuent aux inégalités.

**Méthodes:**

Etude transversale utilisant les données de l'enquête 2014 sur la vie de famille en Indonésie (N = 30.762 pour l'hypertension; N = 6.758 pour le DT2). Le statut socioéconomique a été mesuré par la consommation des ménages. La prévalence de l'hypertension et du DT2 a été déterminée à l'aide de mesures cliniques standardisées à l’échelle internationale, tandis que la prise en charge de la maladie était définie par la participation au dépistage et à l'utilisation courante de médicaments. L'indice relatif d'inégalité (RII) a été utilisé pour estimer les inégalités, ajustées à l’échelle de l’éducation et à la localisation géographique.

**Résultats:**

Pour tous les quintiles de consommation des ménages, nous avons observé de faibles taux de participation au dépistage du DT2 et une faible utilisation de médicaments dans l'hypertension et le DT2. Nous avons constaté des inégalités socioéconomiques dans la participation au dépistage de l'hypertension (RII= 2,68; IC95%: 2,42 à 2,96) et du DT2 (RII= 7,30; IC95%: 5,48 à 9,72), ainsi que de l'utilisation de médicaments pour l'hypertension (IIR= 3,09; IC95%: 2,28 à 4.18) et pour le DT2 (RII= 2,81; IC95%: 1,09–7,27). Le niveau d’éducation a contribué aux inégalités socioéconomiques dans l'utilisation du dépistage pour l'hypertension et le DT2. La localisation géographique a contribué aux inégalités dans l'utilisation du dépistage et de l'utilisation de médicaments pour le DT2. Les inégalités socioéconomiques dans l'utilisation des médicaments contre l'hypertension et le DT2 étaient plus importantes chez les hommes que chez les femmes.

**Conclusions:**

De grandes inégalités socioéconomiques ont été constatées dans l'utilisation des services de prise en charge de l'hypertension et du DT2 en Indonésie. Améliorer l'aspect abordable, la disponibilité et l'accessibilité des services est crucial pour réduire ces inégalités.

## Introduction

The share of non‐communicable diseases (NCDs) in the overall burden of disease in lower middle‐income countries (LMICs) is increasing. WHO has estimated that 63% of deaths worldwide in 2008 were due to NCDs, with about 80% of these occurring in LMICs 1. NCDs cause 56% of deaths in LMICs. In Indonesia, NCDs are responsible for as many as 71% of all deaths. More specifically, cardiovascular diseases, diabetes, cancer and chronic respiratory disease together contribute to 61% of deaths 2.

Proper management of NCDs, including early detection (screening) and the use of cost‐effective medications for people with high‐risk conditions, reduces the burden of NCDs 3. This requires that such services be universally accessible. Many LMICs struggle to provide adequate access to NCD management for all population groups, in particular for lower‐income groups 4. Multi‐country studies have shown that socioeconomic inequalities in the management and prevalence of NCDs exist in high‐income countries (HICs) and in some LMICs 5, 6, 7. The prevalence of NCDs is higher in lower‐socioeconomic groups, creating a sense of urgency for countries to design and implement strategies to safeguard access to NCD management services for those who need it most.

In Indonesia, socioeconomic inequalities in general healthcare use are significant 8. This, combined with the high burden of NCDs, makes the assessment of inequalities in the use of NCD management among different socioeconomic groups highly relevant. No such studies have yet been conducted in Indonesia. Studies to date have focused mainly on assessing socioeconomic inequalities in the prevalence of NCDs, including hypertension and T2D. A study in 2016 shows that low socioeconomic status was a significant predictor for hypertension, whereas a more recent report from WHO suggests that the prevalence of hypertension in Indonesia is similar across socioeconomic groups and that T2D is more prevalent in higher‐socioeconomic groups 9, 10.

In the present study, we assess socioeconomic inequalities in the use of disease management services (screening participation and medication use) for hypertension and T2D in Indonesia. More specifically, we analyse patterns of socioeconomic inequalities in the use of hypertension and T2D management services for the entire Indonesian population, and according to sex. We also assess whether education level and place of residence contribute to socioeconomic inequalities in the use of the services.

## Methods

### Study design and population

This study used a cross‐sectional design with data from the fifth wave of the Indonesia Family Life Survey (IFLS‐5) conducted in 2014. The IFLS has been conducted since 1993 by RAND Corporation (United States). The IFLS‐5 collected data from 13 Indonesian provinces that comprise 83% of the Indonesian population. The data are stored by RAND and can be publicly accessed through its website. The IFLS‐5 was approved by ethical review committees in the United States and Indonesia. More detailed information about the IFLS can be found elsewhere 11.

We included a total of 30 762 individuals (94.9% of the total survey sample) aged 15 or older for whom complete data were available on all our variables of interest. For the analysis of hypertension medication, we used a subsample of 6962 individuals (21.5% of the total sample) who were objectively diagnosed as having hypertension. The IFLS‐5 collected dried blood samples for 7524 individuals, selected in proportion with the geographical and age composition of the survey population 11. Of these, we included 6758 individuals aged 15 or older for whom complete data were available for analysing T2D prevalence and screening participation. For the analysis of T2D medication, we included 485 individuals (6.4% of the total T2D sample) who had been objectively diagnosed with T2D.

### Measurements

We used household consumption as a proxy of socioeconomic status, as it is considered the most valid measurement of household wealth in developing countries 12. Household consumption consisted of spending on food, non‐food consumables, durable goods, education and housing. These counts were aggregated into monthly estimates and adjusted to household size to allow for economies of scale. Household consumption measurement for different areas was adjusted to account for differences in consumer prices by province and by urban *vs*. rural residence. On the basis of this household consumption measure, individuals were grouped into quintiles.

We included education level and geographical characteristics to serve as explanatory, mediator variables in the analyses. We defined education level according to the International Standard Classification of Education (ISCED) 2011 by UNESCO, using data on highest educational attainment to assign individuals to five groups: pre‐primary, primary, lower secondary, upper secondary and tertiary education. Two geographical characteristics were included: place of residence and province. Place of residence was categorised into urban *vs*. rural; provinces were divided into two groups: those in the Java and Bali islands *vs*. provinces in outer islands.

The IFLS‐5 collected blood pressure measurement data based on the American Heart Association standard 13. Hypertension diagnosis was determined according to the 7th Joint National Committee (JNC‐7) classification 14, 15. The means of systolic and diastolic blood pressure in three consecutive measurements were calculated. A mean of ≥140 mmHg systolic and/or ≥90 mmHg diastolic blood pressure was classified as hypertension. Participation in hypertension screening was determined using self‐reports of blood pressure measurement during the past 12 months. Use of hypertension medication was determined among respondents that had been diagnosed with hypertension, using self‐reports of current hypertension drug consumption.

The IFLS‐5 collected HbA1c data using a dried blood sample (DBS), which was converted into whole‐blood equivalent HbA1c. T2D was defined as an HbA1c level ≥ 6.5%, following criteria from the American Diabetes Association 16. Participation in T2D screening was determined using self‐reports of blood glucose measurement during the past 12 months. The use of TD2 medication was determined among respondents that had been diagnosed with T2D, using self‐reports of current diabetes drug consumption.

### Data analysis

We described prevalence rates of disease occurrence, screening participation and medication use for hypertension and T2D for the overall study population, as well as across population groups defined in terms of household consumption quintile, education level and geographical characteristics. The prevalence rates were calculated as the number of cases per 100 persons; these were age–sex standardised using the direct method, with the survey population as the standard population. For disease occurrence and screening participation, we calculated the prevalence rates based on the number of cases in the overall survey sample. For medication use, the prevalence rates were determined by the number of cases among respondents with the disease of interest. On the basis of these prevalence rates, we calculated the rate differences and rate ratios to compare household consumption quintiles, education levels and geographical locations. Rate difference was determined by subtracting the prevalence rate in the highest group (e.g. fifth household consumption quintile) from the rate in the lowest group (e.g. first household consumption quintile). Rate ratio was determined by dividing the prevalence rate in the highest group by that in the lowest group 17.

We used the relative index of inequality (RII) to estimate the magnitude of socioeconomic inequalities in disease prevalence, screening participation and medication use more comprehensively. The RII is a regression‐based index that assesses the prevalence of an outcome measure in relation to the relative position of every individual within the socioeconomic hierarchy. A higher RII value indicates a stronger association between that hierarchical position and the outcome measure. This implies a greater difference in outcome between lower‐ and higher‐socioeconomic groups. Details on how RII scores are calculated can be found elsewhere 18. In the first model, we controlled for age and sex to gauge any associated socioeconomic inequalities. In the second model, we additionally controlled for education level and geographical location to assess any contribution they might make to socioeconomic inequalities.

## Results

We provide the basic characteristics of the study samples for hypertension and T2D in Table 1. The characteristics of the two samples were similar. In general, the proportion of women to men was larger. The 15‐to‐30 age subgroup was the largest of all age groups. The median household consumption of the richest quintile was around five times that of the poorest. The largest subgroups had upper secondary education, lived in urban areas and lived in provinces located on Java or Bali.

**Table 1:** Basic characteristics of the hypertension and type 2 diabetes samples

	Hypertension						Type 2 Diabetes					
	Total		Men		Women		Total		Men		Women	
	*n*	%	*n*	%	*n*	%	*n*	%	*n*	%	*n*	%
Sex												
Men	14393	46.8	–	–	–	–	3030	44.8	–	–	–	–
Women	16333	53.2	–	–	–	–	3728	55.2	–	–	–	–
Age												
15–30	11392	37.1	5133	35.7	6259	38.3	2074	30.7	969	32.0	1105	29.6
31–45	10906	35.5	5258	36.5	5648	34.6	1809	26.8	859	28.3	950	25.5
46–60	5836	19.0	2748	19.1	3088	18.9	1377	20.4	531	17.5	846	22.7
>60	2592	8.4	1254	8.7	1338	8.2	1498	22.2	671	22.1	827	22.2
Household consumption in IDR*												
Quintile 1 (poorest)	1014	20.0	1012	20.0	1016	20.0	949	20.0	949	20.0	949	20.0
Quintile 2	1565	20.0	1566	20.0	1563	20.0	1492	20.0	1501	20.0	1486	20.0
Quintile 3	2161	20.0	2170	20.0	2157	20.0	2062	20.0	2077	20.0	2038	20.0
Quintile 4	3034	20.0	3038	20.0	3032	20.0	2943	20.0	2947	20.0	2938	20.0
Quintile 5 (richest)	5401	20.0	5406	20.0	5394	20.0	5124	20.0	5096	20.0	5139	20.0
Education												
Pre‐primary	5635	18.3	2325	16.2	3310	20.3	1746	25.8	594	19.6	1152	30.9
Primary	6669	21.7	3152	21.9	3517	21.5	1478	21.9	715	23.6	763	20.5
Lower secondary	6597	21.5	3044	21.1	3553	21.8	1229	18.2	564	18.6	665	17.8
Upper secondary	8946	29.1	4575	31.8	4371	26.8	1738	25.7	902	29.8	836	22.4
Tertiary	2879	9.4	1297	9.0	1582	9.7	567	8.4	255	8.4	312	8.4
Location												
Urban	18084	58.9	8442	58.7	9642	59.0	3888	57.5	1747	57.7	2141	57.4
Rural	12642	41.1	5951	41.3	6691	41.0	2870	42.5	1283	42.3	1587	42.6
Provinces												
Java and Bali	16707	54.4	7742	53.8	8965	54.9	3860	57.1	1752	57.8	2108	56.5
Outer islands	14019	45.6	6651	46.2	7368	45.1	2898	42.9	1278	42.2	1620	43.5

*Median household consumption in thousands of Indonesian rupiah (IDR).

The prevalence rates and estimates of socioeconomic inequalities in hypertension are shown in Table 2. Overall, hypertension prevalence was 22.7 per 100 persons and the rate of participation in hypertension screening was 79.0 per 100. For individuals who had been diagnosed with hypertension in terms of the standardised clinical measures in the survey, we found that the use of hypertension medication was 8.1 per 100 persons. Hypertension prevalence was similar among household consumption quintiles, but the richest quintiles had the highest rates of both screening participation and medication use. The rate of medication use was relatively low for all household consumption quintiles, though lowest in the poorest quintile. The highest education group (tertiary education) had the highest rates of hypertension screening participation and medication use. Hypertension prevalence, screening participation and medication use were generally higher in urban areas.

**Table 2:** Estimates of absolute and relative inequalities in the prevalence and the utilisation of disease management services in hypertension

	Disease prevalence		Screening		Medication	
Overall						
SPR (95% CI)*	22.7	22.1–23.2	79.0	78.0–80.0	8.1	7.4–8.8
Household consumption						
SPR (95% CI)						
Quintile 1 (poorest)	22.91	21.76–24.10	72.96	70.81–75.16	5.22	4.18–6.43
Quintile 2	22.11	20.96–23.91	76.80	76.43–79.02	7.75	6.36–9.35
Quintile 3	22.26	21.08–23.50	78.60	76.40–80.85	7.45	6.03–9.11
Quintile 4	22.84	21.61–24.11	81.46	79.21–83.76	9.22	7.60–11.08
Quintile 5 (richest)	22.92	21.69–24.19	85.46	83.14–87.82	11.84	9.97–13.96
Rate difference†	0.01	–	12.50	–	6.62	–
Rate ratio‡	1.00	–	1.17	–	2.27	–
RII (95% CI)§	1.03	0.93–1.14	2.68	2.42–2.96	3.09	2.28–4.18
RII (95% CI)¶	1.12	1.01–1.25	1.67	1.50–1.83	2.41	1.73–3.34
Education						
SPR (95% CI)*						
Pre‐primary	25.68	24.17–27.24	69.87	66.74–73.08	6.65	5.64–7.77
Primary	23.09	21.98–24.25	74.41	72.34–76.52	7.24	5.99–8.68
Lower secondary	20.43	18.95–21.98	78.22	75.73–80.76	10.50	7.90–13.63
Upper secondary	21.81	20.51–23.17	85.41	83.23–87.63	9.69	7.42–12.34
Tertiary	22.13	20.14–24.23	92.51	88.79–96.33	14.66	10.87–19.18
Rate difference**	‐3.55	–	22.64	–	8.01	–
Rate ratio††	0.86	–	1.32	–	2.20	–
OR (95% CI)						
Pre‐primary	1.00	–	1.00	–	1.00	–
Primary	0.89	0.81–0.97	1.67	1.53–1.82	1.04	0.81–1.33
Lower secondary	0.77	0.69–0.85	1.93	1.75–2.13	1.34	0.97–1.84
Upper secondary	0.78	0.71–0.86	3.34	3.03–3.68	1.08	0.80–1.46
Tertiary	0.76	0.67–0.86	6.01	5.12–7.06	1.96	1.40–2.76
Location						
SPR (95% CI)*						
Rural	21.95	21.15–22.77	77.19	76.16–79.25	6.44	5.55–7.43
Urban	23.18	22.47–23.90	79.98	78.68–81.30	9.30	8.38–10.29
Rate difference‡‡	1.23		2.79	–	2.86	–
Rate ratio§§	1.06		1.04	–	1.44	–
OR (95% CI)						
Rural	1.00	–	1.00	–	1.00	–
Urban	1.14	1.06–1.21	0.92	0.87–0.98	1.42	1.17–1.23
Provinces						
SPR (95% CI)*						
Outer islands	21.94	21.15–22.74	79.89	78.41–81.39	7.96	6.96–9.07
Java and Bali	23.26	22.55–23.99	78.36	77.02–79.72	8.15	7.30–9.08
Rate difference¶¶	1.32	–	−1.53	–	0.19	–
Rate ratio***	1.06	–	0.98	–	1.02	–
OR (95% CI)						
Outer islands	1.00	–	1.00	–	1.00	–
Java and Bali	1.08	1.02–1.15	0.91	0.86–0.96	0.99	0.83–1.22

*Standardised prevalence rate per 100 persons with 95% CI, age–sex standardised to the total population.

^†^Difference between richest and poorest quintile.

^‡^Ratio between richest and poorest quintile.

^§^Relative index of inequality, adjusted to age and sex.

^¶^Adjusted to age, sex, education level and geographical areas.

**Difference between tertiary and pre‐primary education level.

^††^Ratio between tertiary and pre‐primary education level.

^‡‡^Difference between urban and rural.

^§§^Ratio between urban and rural.

^¶¶^Difference between Java & Bali and outer islands.

***Ratio between Java & Bali and outer islands.

Measured in terms of the RII, no socioeconomic inequalities in hypertension prevalence were found (RII 1.03, 95% CI 0.93–1.14). However, relatively large socioeconomic inequalities were observed in hypertension screening (RII 2.68, 95% CI 2.42–2.96) and in medication use (RII 3.09, 95% CI 2.28–4.18). The second moderation model showed that the inclusion of education level and geographical factors reduced the RII values, particularly for hypertension screening (RII 1.67, 95% CI 1.50–1.93), indicating a substantial contribution of those factors to socioeconomic inequalities in hypertension screening.

Sex‐stratified socioeconomic inequalities in the utilisation of hypertension management services are estimated in Table 3. Women had higher rates of hypertension screening and medication use as compared to men, findings that were consistent across household consumption quintiles. Only in the use of hypertension medication were socioeconomic inequalities larger for men (RII 4.74, 95% CI 2.73–8.22) than for women (RII 2.63, 95% CI 1.80–3.84).

**Table 3:** Estimates of absolute and relative inequalities in the utilisation of hypertension management services, stratified by sex

	Screening				Medication			
	Men		Women		Men		Women	
Overall								
SPR (95% CI)*	71.6	70.2–73.0	85.5	84.1–87.70	5.3	4.5–6.1	10.6	9.6–11.7
Household consumption								
SPR (95% CI)*								
Quintile 1 (poorest)	64.22	61.30–67.24	80.66	77.56–83.86	2.90	1.78–4.42	7.34	5.56–9.51
Quintile 2	68.80	65.77–71.94	83.85	80.77–87.02	3.75	2.38–5.63	10.72	8.54–13.29
Quintile 3	69.76	66.73–72.90	86.39	83.23–89.63	5.42	3.73–7.62	8.65	6.62–11.10
Quintile 4	74.75	71.63–77.97	87.37	84.17–90.67	5.60	3.91–7.76	12.24	9.75–15.17
Quintile 5 (richest)	80.36	77.12–83.70	89.95	86.67–93.31	8.58	6.48–11.14	15.21	12.28–18.61
Rate difference†	16.14	–	9.29	–	5.68	–	7.87	–
Rate ratio‡	1.25	–	1.12	–	2.96	–	2.07	–
RII (95% CI)§	2.66	2.34–3.03	2.71	2.32–3.17	4.74	2.73–8.22	2.63	1.80–3.84

*Standardised prevalence rate per 100 persons with 95% CI, age‐standardised to the total population.

^†^Difference between richest and poorest quintile.

^‡^Ratio between richest and poorest quintile.

^§^Relative index of inequality, adjusted to age.

The prevalence rates and inequality estimates pertaining to type 2 diabetes (T2D) are shown in Table 4. The overall rate of T2D prevalence was 7.2 per 100 persons, and the rate of T2D screening was 11.2 per 100. For individuals who had been diagnosed with T2D in terms of the standardised clinical measures used in the survey, we found that the rate of T2D medication was 13.0 per 100 persons. The rates of T2D prevalence, screening and medication increased from the poorest to the richest quintiles.

**Table 4:** Estimates of absolute and relative inequalities in the prevalence and the utilisation of disease management services in type 2 diabetes

	Disease prevalence		Screening		Medication	
Overall						
SPR (95% CI)*	7.2	6.6–7.8	11.2	10.4–12.0	13.0	10.0–16.6
Household Consumption						
SPR (95% CI)						
Quintile 1 (poorest)	4.41	3.38–5.66	4.73	3.62–6.07	2.41	0.22–8.93
Quintile 2	7.94	6.52–9.57	8.28	6.83–9.94	15.86	9.23–35.32
Quintile 3	5.52	4.32–6.94	10.39	8.72–12.28	10.80	3.66–23.47
Quintile 4	8.29	6.80–10.01	13.03	11.16–15.13	15.72	8.66–26.01
Quintile 5 (richest)	10.03	8.33–11.96	20.48	18.03–20.17	16.99	10.19–26.50
Rate difference†	5.62	–	15.75	–	14.58	–
Rate ratio‡	2.27	–	4.33	–	7.05	–
RII (95% CI)§	2.41	1.73–3.34	7.30	5.48–9.72	2.81	1.09–7.27
RII (95% CI)¶	2.41	1.71–3.41	3.52	2.65–4.66	1.85	0.64–5.36
Education						
SPR (95% CI)*						
Pre‐primary	8.30	6.32–10.53	4.84	3.85–5.98	9.67	5.26–19.56
Primary	7.09	5.80–8.58	7.10	5.86–8.52	10.29	5.07–18.53
Lower secondary	8.88	6.54–11.68	12.84	9.94–16.32	22.95	11.28–41.35
Upper secondary	8.70	6.76–10.95	20.09	16.96–23.54	14.44	6.58–26.21
Tertiary	7.70	4.62–11.65	37.24	30.10–45.23	35.30	12.08–73.73
Rate difference**	−0.60	–	32.40	–	25.63	
Rate ratio††	0.93	–	7.69	–	3.65	
OR (95% CI)						
Pre‐primary	1.00	–	1.00	–	1.00	
Primary	1.01	0.77–1.32	1.41	1.07–1.86	1.02	0.43–2.39
Lower secondary	1.01	0.72–1.42	2.24	1.63–3.08	2.42	0.98–6.01
Upper secondary	0.98	0.72–1.34	3.52	2.65–4.66	1.58	0.63–3.98
Tertiary	0.80	0.53–1.21	7.87	5.77–10.75	3.92	1.28–11.98
Location						
SPR (95% CI)*						
Rural	6.75	5.84–7.66	5.98	5.12–6.94	6.77	3.27–12.29
Urban	7.52	6.67–8.44	15.15	13.94–16.44	16.56	12.29–21.80
Rate difference‡‡	0.77	–	9.17	–	9.79	–
Rate ratio§§	1.11	–	2.53	–	2.45	–
OR (95% CI)						
Rural	1.00	–	1.00	–	1.00	–
Urban	1.15	0.95–1.41	2.14	1.77–2.58	2.96	1.42–4.16
Provinces						
SPR (95% CI)*						
Outer islands	8.03	7.02–9.15	9.65	8.53–10.87	10.55	6.50–16.17
Java and Bali	6.49	5.72–7.34	12.25	11.18–13.40	15.08	10.84–20.42
Rate difference^¶^^¶^	−1.54	–	2.60	–	4.53	–
Rate ratio***	0.81	–	1.27	–	1.43	–
OR (95% CI)						
Outer islands	1.00	–	1.00	–	1.00	–
Java and Bali	0.81	0.68–0.99	1.27	1.08–1.51	1.59	0.86–2.93

*Standardised prevalence rate per 100 persons, age–sex standardised to the total population.

^†^Difference between richest and poorest quintile.

^‡^Ratio between richest and poorest quintile.

^§^Relative index of inequality, adjusted to age and sex.

^¶^Adjusted to age, sex, education level and geographical areas.

**Difference between tertiary and pre‐primary education level.

^††^Ratio between tertiary and pre‐primary education level.

^‡‡^Difference between urban and rural.

^§§^Ratio between urban and rural.

^¶¶^Difference between Java & Bali and outer islands

***Ratio between Java & Bali and outer islands.

T2D prevalence was lower at the highest education level than at the lowest one, while the rate of T2D screening and medication was highest at  the tertiary level than at other levels. Urban areas showed higher T2D prevalence, screening and medication use than rural areas. Provinces in outer islands had a higher T2D prevalence than those in Java and Bali, while provinces in Java and Bali had higher rates of T2D screening and medication use.

Socioeconomic inequalities were found in terms of T2D prevalence (RII 2.41, 95% CI 1.73–3.34), use of T2D screening (RII 7.30, 95% CI 5.48–9.72) and use of T2D medication (RII 2.81, 95% CI 1.09–7.27). In the second moderation model, the inclusion of education and geographical factors substantially reduced the RII values, and particularly for T2D screening (RII 3.52, 95% CI 2.65–4.66). This indicates an essential contribution of these factors to the socioeconomic inequalities in T2D screening.

After stratification by sex (Table 5), the rates of T2D screening and medication use were similar for men and women. The rates of T2D screening increased linearly across household consumption quintiles for both sexes. The difference between men and women in terms of socioeconomic inequalities in T2D screening participation was relatively modest. However, in the use of T2D medication, men showed larger socioeconomic inequalities than women.

**Table 5:** Estimates of absolute and relative inequalities in type 2 diabetes management, stratified by sex

	Screening				Medication			
	Men		Women		Men		Women	
Overall								
SPR (95% CI)*	11.00	9.9–12.3	11.3	10.2–12.4	12.8	8.2–19.1	13.1	9.3–17.9
Household consumption								
SPR (95% CI)*								
Quintile 1 (poorest)	4.98	3.34–7.14	4.53	3.10–6.38	0.00	–	3.93	0.36–14.53
Quintile 2	7.18	5.22–9.61	9.17	7.15–11.59	10.28	3.21–24.25	19.37	10.07–33.49
Quintile 3	9.56	7.25–12.38	11.06	8.76–13.77	15.61	2.00–48.60	7.78	2.07–20.02
Quintile 4	13.59	10.72–16.76	12.66	10.19–15.54	15.08	6.35–29.98	16.13	6.84–31.99
Quintile 5 (richest)	20.94	17.27–25.15	20.12	16.90–23.75	17.47	6.96–35.41	16.69	8.56–29.26
Rate difference†	15.96	–	15.59	–	17.47	–	12.76	–
Rate ratio‡	4.20	–	4.44	–	n.a	–	4.25	–
RII (95% CI)§	8.12	5.25–12.55	6.71	4.58–9.83	9.27	1.57–54.57	1.83	0.57–5.86

n.a, not applicable.

*Standardised prevalence rate per 100 persons with 95% CI, age‐standardised to the total population.

^†^Difference between richest and poorest quintile.

^‡^Ratio between richest and poorest quintile.

^§^Relative index of inequality, adjusted to age.

## Discussion

This study aimed to assess socioeconomic inequalities in the utilisation of hypertension and T2D management services in Indonesia, to identify any sex differences in inequality patterns, and to assess any contributions of educational and geographical factors to inequalities. We observed no socioeconomic inequalities for hypertension prevalence but relatively large inequalities for T2D prevalence. Relatively large socioeconomic inequalities were also found in the use of hypertension and T2D screening and in the use of corresponding medications. Substantial sex differences in socioeconomic inequalities were found in the use of hypertension and T2D medications, with men showing greater inequalities than women. Education level substantially contributed to inequalities in the use of hypertension and T2D screening. Urban residency contributed to inequalities in T2D screening and medication use.

To our knowledge, this is the first study to comprehensively assess socioeconomic inequalities in the utilisation of hypertension and T2D management services in Indonesia. We used recent, nationally representative data from a large survey sample. Disease prevalence was determined using standardised measurements that conformed with international standards.

We acknowledge limitations in our study. First, the sample size for T2D was relatively small, which reduced the power of that analysis and may have led to false‐negative results. Due to cost constraints, the IFLS‐5 took blood samples from a limited number of individuals. These were selected using several criteria, including the age composition and geographical location of the survey population, in order to ensure the representativeness of this subsample 11. Second, we used self‐reported data for screening history and current medication use, thus raising the possibility of recall bias in our data; in the absence of national registry‐based data, however, self‐reported data obtained with a validated survey questionnaire were the best available data source. Third, our measure of household consumption did not include some types of spending, such as healthcare and transportation costs, and this may influence the accuracy of our estimations. Any inaccuracies are probably small, however, as we adhered to the method suggested by the IFLS‐5 guidelines, thus capturing most major types of household spending.

A notable finding in our study involves the relatively low rates in all household consumption quintiles in terms of screening for T2D, medication use for T2D and medication use for hypertension. This finding indicates a ‘total population problem’, which may be attributable to system‐wide inadequacies in the healthcare system. Studies have shown that countries that provide specific NCD management services, such as nationwide screening and case management, achieve better management coverage 19, 20. Indonesia lacks such national screening programmes and also effective case management approach for long‐term hypertension and T2D treatment particularly at primary care level 21. The lack of screening applies in particular to T2D, which requires specific laboratory testing to confirm diagnosis. For hypertension, only blood pressure measurements are required to confirm diagnosis, a procedure routinely conducted when patients visit healthcare facilities, regardless of the underlying clinical condition. This likely led to the much higher rates of hypertension screening than T2D testing.

The socioeconomic inequalities we observed in the utilisation of hypertension and T2D management services in Indonesia are consistent with findings on socioeconomic inequalities in access to NCD‐related healthcare services in HICs and some LMICs 5, 7, 22. Our results can be interpreted in relation to three important, interrelated dimensions in access to health care: the approachability, availability and affordability of services 23.

First, the *approachability* of health care involves people's possibilities to become aware that a relevant healthcare service exists and can be accessed 23. As mentioned, the lack of nationwide systematic hypertension and T2D services in Indonesia may lead to lack of information on the existence of services that are relevant to people's own health 21. This may particularly affect those with lower health literacy, less health‐related knowledge or specific disease beliefs that inhibit the search for services. In this connection, it is important to note that the socioeconomic inequalities we found in the utilisation of hypertension and T2D management services, and particularly in disease screening, substantially diminished after adjustment for education level. Highly educated people generally have better health knowledge and access to health information and a greater propensity to seek timely access to services 24. Several other studies have found that education level substantially influenced the use of healthcare services for NCDs, particularly in terms of preventive care, and that such influence was independent of income level 19, 25, 26, 27.

Second, the *availability* of health care relates to the geographical location of facilities, which may determine opportunities and costs for their use 23. We observed that people living in urban areas were more likely to use T2D management services compared with those in rural areas. To a lesser extent, we found similar differences when Java and Bali were compared to outer islands. These findings may relate to an unequal distribution of healthcare facilities among geographical areas in Indonesia 10. More specifically, as T2D management requires more sophisticated services like laboratory examination and specific medication, the availability of such services may be inadequate in rural or remote areas.

Third, the *affordability* of health care is dependent on the economic capacity of an individual to cope with healthcare‐related costs, including direct costs, indirect costs and opportunity costs 23. The direct costs are unlikely to have been a major contributing factor, as such costs would have been covered by the Indonesian National Health Insurance (NHI) programme, particularly in lower‐socioeconomic groups. Indirect and opportunity costs, on the other hand, may have contributed to socioeconomic inequalities for several reasons, as hypertension and T2D are chronic diseases that require frequent, long‐term contacts between individuals and healthcare providers. Such frequent health service use may entail considerable indirect costs and opportunity costs, which higher‐socioeconomic groups have more economic capacity to cope with.

We observed that socioeconomic inequalities in medication use for both hypertension and T2D were wider among men than among women. It is well‐documented that men exhibit different healthcare‐seeking behaviours and use less health care than women. Such behaviour is widely attributed to the concept of hegemonic masculinity 28, whereby men may believe that seeking help for a particular condition is appropriate only when it is serious enough and help‐seeking is deemed acceptable by society. As a result, men may tend to ‘normalise’ their health and to delay seeking care until a more advanced stage of illness. Such behaviour may vary by socioeconomic status, with men of low SES tending to have stronger masculine identities, particularly with regard to preventive care 28, 29, 30.

To conclude, our findings confirm the existence of socioeconomic inequalities in the utilisation of hypertension and T2D management services in Indonesia. A nationwide systematic hypertension and T2D management strategy is urgently needed to address the low coverage of hypertension and T2D services in all population strata. Increasing the availability and the proximity of services could facilitate access to hypertension and T2D management services for lower‐socioeconomic groups. Affordability of services could be improved by reducing the indirect and opportunity costs of hypertension‐ and T2D‐related health care. Improving the approachability of health care by developing more regular outreach activities and more effective communication strategies could help resolve the education‐related problems in access to hypertension and T2D management services. Such a strategy could be part of a wider agenda to improve the health literacy of the population.

## References

[1] Alwan A . Global Status Report on Noncommunicable Diseases 2010. World Health Organization: Geneva; 2011.

[2] Riley LM , Cowan M . Noncommunicable Diseases Country Profile 2014. World Health Organization: Geneva, 2014.

[3] Prevention and Control of Noncommunicable Diseases . Guidelines for Primary Health Care in Low Resource Settings. World Health Organization: Geneva, 2012. 23844451

[4] El‐Sayed AM , Palma A , Freedman LP , Kruk ME . Does health insurance mitigate inequities in non‐communicable disease treatment? Evidence from 48 low‐ and middle‐income countries. Health Policy 2015: 119: 1164–1175. 26271138 10.1016/j.healthpol.2015.07.006 PMC5927367

[5] Christiani Y , Dhippayom T , Chaiyakunapruk N . Assessing evidence of inequalities in access to medication for diabetic populations in low‐ and middle‐income countries: a systematic review. Glob Health Action 2016: 9: 32505. 27938647 10.3402/gha.v9.32505 PMC5148807

[6] Gakidou E , Mallinger L , Abbott‐Klafter J *et al* Management of diabetes and associated cardiovascular risk factors in seven countries: a comparison of data from national health examination surveys. Bull World Health Organ 2011: 89: 172–183. 21379413 10.2471/BLT.10.080820 PMC3044248

[7] Palafox B , McKee M , Balabanova D *et al* Wealth and cardiovascular health: a cross‐sectional study of wealth‐related inequalities in the awareness, treatment and control of hypertension in high‐, middle‐ and low‐income countries. Int J Equity Health 2016: 15: 199. 27931255 10.1186/s12939-016-0478-6 PMC5146857

[8] Mulyanto J , Kringos DS , Kunst AE . Socioeconomic inequalities in healthcare utilisation in Indonesia: a comprehensive survey‐based overview. BMJ Open 2019: 9: e026164. 10.1136/bmjopen-2018-026164 PMC6661624 31326926

[9] Hussain MA , Mamun AA , Reid C , Huxley RR . Prevalence, awareness, treatment and control of hypertension in indonesian adults aged ≥ 40 years: findings from the Indonesia Family Life Survey (IFLS). PLoS ONE 2016: 11: e0160922. 27556532 10.1371/journal.pone.0160922 PMC4996427

[10] Hosseinpoor AR , Bergen N , Floranita R , Kusumawardani N , Schlotheuber A . State of Health Inequality: Indonesia. World Health Organization: Geneva, 2017.

[11] Strauss J , Witoelar F , Sikoki B . The Fifth Wave of the Indonesia Family Life Survey: Overview and Field Report. RAND Labor and Population; 2016. Contract No.: WR‐1143/1‐NIA/NICHD

[12] O'Donnel O , Doorslaer EV , Wagstaff A , Lindelow M . Analyzing Health Equity Using Household Survey Data: A Guide to Techniques and Their Implementation. Washington: The World Bank Institute; 2008.

[13] Pickering TG , Hall JE , Appel LJ *et al* Recommendations for blood pressure measurement in humans and experimental animals. Part 1: Blood pressure measurement in humans: a statement for professionals from the Subcommittee of Professional and Public Education of the American Heart Association Council on High Blood Pressure. Hypertension 2005: 45: 142–161. 15611362 10.1161/01.HYP.0000150859.47929.8e

[14] Chobanian AV , Bakris GL , Black HR *et al* The seventh report of the joint national committee on prevention, detection, evaluation, and treatment of high blood pressure: the JNC 7 report. JAMA 2003: 289: 2560–2571. 12748199 10.1001/jama.289.19.2560

[15] James PA , Oparil S , Carter BL *et al* 2014 evidence‐based guideline for the management of high blood pressure in adults: report from the panel members appointed to the Eighth Joint National Committee (JNC 8). JAMA 2014: 311: 507–520. 24352797 10.1001/jama.2013.284427

[16] American Diabetes Association . Classification and diagnosis of diabetes. Sec. 2. In Standards of Medical Care in Diabetesd 2015. Diabetes Care 2015: 38(Suppl. 1): S8–S16. 10.2337/dc15-S005 25537714

[17] Fleiss JL , Levin B , Paik MC . Statistical Methods for Rates and Proportions (3rd edn), John Wiley & Sons, Inc.: Hoboken, NJ, 2003.

[18] Mackenbach JP , Kunst AE . Measuring the magnitude of socio‐economic inequalities in health: an overview of available measures illustrated with two examples from Europe. Soc Sci Med 1997: 44: 757–771. 9080560 10.1016/s0277-9536(96)00073-1

[19] Rodin D , Stirbu I , Ekholm O *et al* Educational inequalities in blood pressure and cholesterol screening in nine European countries. J Epidemiol Community Health 2012: 66: 1050–1055. 22245720 10.1136/jech-2011-200273

[20] Palència L , Espelt A , Rodríguez‐Sanz M *et al* Socio‐economic inequalities in breast and cervical cancer screening practices in Europe: influence of the type of screening program. Int J Epidemiol 2010: 39: 757–765. 20176587 10.1093/ije/dyq003

[21] Schröders J , Wall S , Hakimi M *et al* How is Indonesia coping with its epidemic of chronic noncommunicable diseases? A systematic review with meta‐analysis. PLoS ONE 2017: 12: e0179186. 28632767 10.1371/journal.pone.0179186 PMC5478110

[22] Ricci‐Cabello I , Ruiz‐Perez I , Olry de Labry‐Lima A , Marquez‐Calderon S . Do social inequalities exist in terms of the prevention, diagnosis, treatment, control and monitoring of diabetes? A systematic review. Health Soc Care Community 2010: 18: 572–587. 21040063 10.1111/j.1365-2524.2010.00960.x

[23] Levesque J‐F , Harris MF , Russell G . Patient‐centred access to health care: conceptualising access at the interface of health systems and populations. Int J Equity Health 2013: 12: 18. 23496984 10.1186/1475-9276-12-18 PMC3610159

[24] Rosenstock IM . Why people use health services. Milbank Q 2005: 83:1–32.

[25] Katz SJ , Hofer TP . Socioeconomic disparities in preventive care persist despite universal coverage: breast and cervical cancer screening in Ontario and the United States. JAMA 1994: 272: 530–534. 8046807

[26] Hsu CC , Lee CH , Wahlqvist ML *et al* Poverty increases type 2 diabetes incidence and inequality of care despite universal health coverage. Diabetes Care 2012: 35: 2286–2292. 22912425 10.2337/dc11-2052 PMC3476930

[27] Ahmed SM , Tomson G , Petzold M , Kabir ZN . Socioeconomic status overrides age and gender in determining health‐seeking behaviour in rural Bangladesh. Bull World Health Organ 2005: 83: 109–117. 15744403 PMC2623805

[28] Galdas PM , Cheater F , Marshall P . Men and health help‐seeking behaviour: literature review. J Adv Nurs 2005: 49: 616–623. 15737222 10.1111/j.1365-2648.2004.03331.x

[29] Noone JH , Stephens C . Men, masculine identities, and health care utilisation. Sociol Health Illn 2008: 30: 711–725. 18564976 10.1111/j.1467-9566.2008.01095.x

[30] Springer KW , Mouzon DM . “Macho Men” and preventive health care: implications for older men in different social classes. J Health Soc Behav 2011: 52: 212–227. 21490311 10.1177/0022146510393972

